# Navigating the bridge between wet and dry lab toxicology research to address current challenges with high-dimensional data

**DOI:** 10.3389/ftox.2023.1171175

**Published:** 2023-05-26

**Authors:** Alexis Payton, Kyle R. Roell, Meghan E. Rebuli, William Valdar, Ilona Jaspers, Julia E. Rager

**Affiliations:** ^1^ Department of Environmental Sciences and Engineering, Gillings School of Global Public Health, University of North Carolina at Chapel Hill, Chapel Hill, NC, United States; ^2^ Center for Environmental Medicine, Asthma and Lung Biology, School of Medicine, University of North Carolina at Chapel Hill, Chapel Hill, NC, United States; ^3^ The Institute for Environmental Health Solutions, Gillings School of Global Public Health, University of North Carolina at Chapel Hill, Chapel Hill, NC, United States; ^4^ Curriculum in Toxicology and Environmental Medicine, School of Medicine, University of North Carolina at Chapel Hill, Chapel Hill, NC, United States; ^5^ Department of Pediatrics, School of Medicine, University of North Carolina at Chapel Hill, Chapel Hill, NC, United States; ^6^ Department of Genetics, University of North Carolina, Chapel Hill, NC, United States

**Keywords:** computational toxicology, data analysis, machine learning, high dimensional data, data imputation

## Abstract

Toxicology research has rapidly evolved, leveraging increasingly advanced technologies in high-throughput approaches to yield important information on toxicological mechanisms and health outcomes. Data produced through toxicology studies are consequently becoming larger, often producing high-dimensional data. These types of data hold promise for imparting new knowledge, yet inherently have complexities causing them to be a rate-limiting element for researchers, particularly those that are housed in “wet lab” settings (i.e., researchers that use liquids to analyze various chemicals and biomarkers as opposed to more computationally focused, “dry lab” researchers). These types of challenges represent topics of ongoing conversation amongst our team and researchers in the field. The aim of this perspective is to i) summarize hurdles in analyzing high-dimensional data in toxicology that require improved training and translation for wet lab researchers, ii) highlight example methods that have aided in translating data analysis techniques to wet lab researchers; and iii) describe challenges that remain to be effectively addressed, to date, in toxicology research. Specific aspects include methodologies that could be introduced to wet lab researchers, including data pre-processing, machine learning, and data reduction. Current challenges discussed include model interpretability, study biases, and data analysis training. Example efforts implemented to translate these data analysis techniques are also mentioned, including online data analysis resources and hands-on workshops. Questions are also posed to continue conversation in the toxicology community. Contents of this perspective represent timely issues broadly occurring in the fields of bioinformatics and toxicology that require ongoing dialogue between wet and dry lab researchers.

## 1 Introduction to current problems in analyzing toxicology data

**FIGURE 1:**
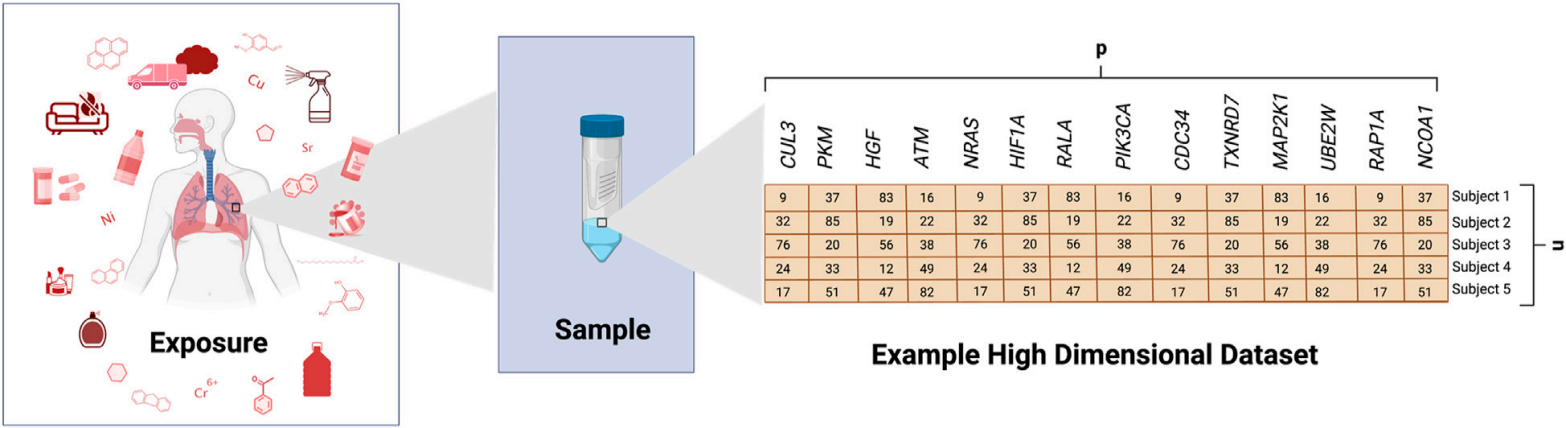
Generation of an Example High-Dimensional Dataset. This example illustrates some potential exposure conditions that may be evaluated in toxicology studies. Biological samples are shown as collected from human subjects and analyzed for gene expression levels across several genes, as an example (generic variable, *p*). This figure shows an example where samples were collected across 5 subjects (*n*), resulting in a high-dimensional dataset with *p* >> *n*.

It is now routine for toxicology studies to investigate many biological and chemical endpoints at once, representing an advancement occurring over the past decade resulting in drastic changes to the field (Rager et al, 2013; Sobus et al, 2018). Such research includes *in vitro*, animal, and/or clinical studies designed to ultimately evaluate stressor-associated hazards, risk profiles, and intervention efforts. Technologies continue to advance, allowing for increasingly high-dimensional measures; though we remain limited by the number of samples (i.e., cell or animal samples, or human subjects) that can be feasibly evaluated in such investigations. As a result, the field of toxicology is currently plagued with the following data analysis problems (Sobus et al, 2018): we need to better analyze large datasets, including high-dimensional data (Figure 1) and (Rager et al, 2013) we need to better advise and train “wet lab” scientists to analyze such dataset. Although there continues to be continued discussion of what constitutes big data and high-dimensional data, big data can refer to data that challenges existing methods of computational applications as a result of its size, complexity, or rate of availability (Favaretto et al, 2020). High-dimensional data refers to data where the number of variables (*p*) is larger than the number of samples/subjects (*n*) (*p >> n*)*.* However, it can also refer to *p* and *n* both being substantially large or even *p* << *n* (Finney, 1977). This problem represents an issue that must be addressed to keep up with data currently being generated in toxicology research. Related to this issue is how to identify relationships between a multitude of often highly correlated predictor variables and one or a few outcomes of interest. Due to its size, big data, including those with high-dimensionality, can be difficult to process, store, transfer, analyze, and visualize. However, methods to address this and other problems (i.e., missing data, interpretability, etc.) arising in the realm of high-dimensional data are rapidly expanding in other fields and are being adapted to toxicological research.

### 1.2 Aim and organization of perspective article

The aim of this perspective is to i) draw attention to the current need to improve data analyses for large datasets, ii) highlight some example methods of organizing and analyzing high-dimensional data, and iii) describe challenges that now need to be addressed to improve data analyses in toxicology research, all of which represent issues of current discussion between wet lab and dry lab scientists. Examples are relevant to high-dimensional data issues that are prevalent in data produced through toxicology research. The contents of this perspective originated from discussions held in the University of North Carolina’s newly formed Environmental Bioinformatics Research Group (EBioRG) (Environmental Bioinformatics Research Group EBRG, 2023), representing timely communications among data scientists, computational biologists, physician-scientists, and experimental toxicologists akin to those occurring across other research groups world-wide surrounding the improved analysis of data in the field of toxicology.

This perspective is organized to first provide a high-level overview of current data analysis methods for large datasets, including data preparations and downstream machine learning (ML) methods discussed in the context of recent toxicology-relevant studies. The second part of the perspective then discusses current limitations, biases, and a push for the inclusion of these methods in toxicology. Questions are posed in the final section to encourage continued discussion on this evolving subject.

## 2 Prepping high-dimensional data to optimize downstream analyses through data Imputation

There are many steps involved in preparing high dimensional data for analyses. One of these steps represents a common issue that scientists face when collecting data in the wet lab: data may be missing due to different laboratory-based limitations including detection limits, experimental errors, etc. In these datasets, retaining observations (i.e., samples, subjects, or endpoints) with missing data helps *n* be less prone to skew and therefore minimizes bias in downstream analyses. This is particularly important when dealing with high-dimensional data that suffers from a limited number of observations. Data imputation is a commonly used technique to generate these missing values based on the statistical distribution of present/detected experimental values. Prior to imputation, background filters can be implemented to filter poorly measured or highly missing variables and observations to ensure that enough data is present for the imputed data to be pulled from a data-informed distribution. After these filters are implemented, an evaluation should be carried out by the analysts to consider why data are missing. Current categorizations of missing data include missing completely at random (MCAR), missing at random (MAR), or missing not at random (MNAR) (Burren, 2018). Data are considered to be MCAR if the probability of an observation is independent of observed or unobserved data. This could occur if a sample was damaged in the lab. Data are MAR when missingness is dependent upon only the observed data (Bhaskaran and Smeeth, 2014). This could happen if a demographic group is oversampled in a study. Lastly, MNAR values are generated when missingness is dependent upon unobserved data (Burren, 2018). For transcriptomics, proteomics, metabolomics, and exposomics data, left-censored data MNAR are often the result of low and/or no expression of the gene, protein, molecule, or chemical, respectively.

The type of missing data imputation approach should be informed by the reasoning as to why data may be missing. As an example, a metabolomics dataset was analyzed in a previous toxicology-relevant study, and missing data were imputed using eight different imputation methods spanning assumptions of MCAR, MAR, and MNAR (Wei et al, 2018). One of the evaluated imputation methods was based upon random forest (RF) typically used to generate values for variables presumed to be MCAR or MAR across the entirety of the data distribution. Another method was evaluated, namely, the Quantile Regression Imputation of Left-Censored data (QRILC) method which is typically used to impute MNAR values near or well below the lowest detected expression level from the left side of a Gaussian distribution. In this case study, RF outperformed all other methods for MCAR/MAR imputation, while QRILC performed best for MNAR imputation (Wei et al, 2018). Although RF has rapidly gained acceptance as a routine technique and its high performance has been replicated in other studies (Waljee et al, 2013; Tang and Ishwaran, 2017; Ramosaj and Pauly, 2019), it has also been shown that RF-based imputation on highly skewed MAR clinical data can bias subsequent regression analyses (Hong and Lynn, 2020). Data imputation methods have been examined further in review studies (Cummings, 2013; Allotey and Harel, 2019; Patruno et al, 2021). These findings illustrate that imputation should not have a blanket approach and highlights the importance of taking into consideration the type of data being imputed during the pre-processing of high-dimensional data. Other elements of prepping high-dimensional data for analyses include, but are not limited to, data normalization, identification of potential sample outliers, and detection of batch effects.

## 3 Current example machine learning approaches for analyzing high-dimensional data in toxicology research

### 3.1 Predicting toxicological endpoints using supervised ML

Data produced through wet lab experimentation are often analyzed using traditional statistics based on two-group comparisons; though if given the tools and guidance, these data could be analyzed using machine learning (ML) to unravel undiscovered biological patterns. ML automates the building of analytical models from parameters tuned by the researcher based on the idea that it can learn from data, identify patterns, and make decisions utilizing multiple independent variables (SAS, 2023). Supervised ML is one category of ML that learns how to predict a labeled outcome from a training dataset. Specifically, it first iteratively makes predictions of the labeled outcome using a large portion of a dataset (termed the “training set”) with the algorithm seeking to predict as accurately as possible. The trained model is then applied to the remaining portion of the dataset or a separate dataset (termed the “test set”) to determine how well it performs at predicting data it has not seen before (Green et al, 2021). Most supervised ML algorithms require a vast amount of training data and number of observations (*n*), which may not be feasible for all toxicological experiments. Note, there is not a “magic formula” for determining the optimal *n* to perform supervised ML, however it is contingent upon the number of features (*p*) and the number of classes of a labeled outcome being predicted. Nevertheless, supervised ML is particularly useful for large datasets in toxicology given its ability to predict a toxicological outcome from a multitude of features (e.g., -omic signatures, demographic variables, etc.).

One supervised ML algorithm, RF has grown in popularity due to its ability to use decision trees to determine the probability of a predicted outcome by assessing one predictor at a time. RF, like many supervised ML models, can also include data with varying distributions, making it highly adaptable under many scenarios (Koutsoukas et al, 2016; Green et al, 2021). RF models have been built to predict many types of toxicological outcomes leveraging high-dimensional data as predictor variables. Examples include studies that have utilized multi-omic signatures to predict many different health outcomes, including birth outcomes (Koutsoukas et al, 2016); cancer metastasis (Albaradei et al, 2021); and suicide risk (Bhak et al, 2019). *In vitro* high-throughput screening data have also been used in RF models to predict multiple *in vivo* outcomes, such as liver pathway responses (Ring et al, 2021), liver lesions (Liu et al, 2017), neuroactivity patterns (Kosnik et al, 2020), and developmental toxicity (Sipes et al, 2011). These studies, including review studies, demonstrate the utility of supervised ML methods when incorporating high-dimensional data into toxicological research (Ekins, 2014; Omer et al, 2014; Idakwo et al, 2018).

### 3.2 Discovering patterns in high-dimensional data using unsupervised ML

Approaches that can also be used to better evaluate data produced through wet lab experimentation include unsupervised ML. Supervised ML is suitable if the outcome or labels are known, but what if the collected data has unknown outcomes or labels? In these instances, unsupervised ML methods can be leveraged, which seek to find associations between data that lack defined classifications or labels (IBM, 2020). Given the vast array of toxicants yet to be fully analyzed, unsupervised ML can be a productive first step. For instance, it can uncover potential patterns between biomarkers, toxicants, environmental, and demographic factors, along with other stressors that can influence biological outcomes.

One unsupervised ML algorithm, k-means, groups observations into the optimal number of clusters with each observation belonging to the clusters with the closest mean (Ecosystem, 2018). As an example, an inhalation toxicology study carried out by our group evaluated cytokine signatures derived from multiple biological samples that commonly inform respiratory responses, including nasal lavage fluid, nasal epithelial lining fluid, sputum, and circulating serum, to determine if cytokine levels differed based on these sampling locations (Payton et al, 2022). K-means grouped cytokines into clusters within each respiratory tract region, separately. These groupings revealed that cytokine signatures are dependent upon sampling location, informing the future importance of such biomarkers in the clinical setting (Payton et al, 2022).

Another unsupervised ML technique that seeks to find inferences between data is hierarchical clustering. Hierarchical clustering works by initially treating each observation as its own cluster and merges them into larger clusters based on the minimum, maximum, or average distance between observations (Bock, 2022). A recent study leveraged hierarchical clustering to identify understudied environmental compounds that humans are likely co-exposed to alongside chemicals known to cause breast cancer (Koval et al, 2022). Findings were of particular importance given these chemicals are known to be present in personal care, food, and toy products, to name a few. Hierarchical clustering also found that some of the understudied chemicals share similar chemical properties with cancer-associated compounds (Koval et al, 2022). In general, clustering analyses like k-means and hierarchical clustering allow researchers to draw relationships between variables and select specific variables that might warrant further investigation, potentially reducing the size of a high-dimensional dataset in future studies. These approaches can significantly inform further environmental and biological meaning behind data currently being produced throughout toxicological wet lab experimentation.

### 3.3 Reducing the dimensionality of toxicological data using unsupervised ML

There are many instances when analyzing large datasets where reducing the number of predictors into aggregate values can aid in identifying relationships that may not exist or be apparent when analyzing all variables individually. Known as dimensionality reduction, this technique is useful for analyzing high-dimensional datasets that are more difficult to visualize, analyze, and require a longer amount of computing time (Ian and Johnstone, 2009; Yiu, 2019). Principal Component Analysis (PCA) has become a widely adopted computational tool that seeks to address this issue by compressing as much of the variance from the original dataset into the fewest number of principal components or eigenvectors. These eigenvectors serve to be representative of the original variables with the first accounting for most of the variance that tapers off with each successive eigenvector (Furihata et al, 2016). Although PCA is another unsupervised ML method, as with k-means and hierarchical clustering, it compresses most of a dataset’s variance within the first few principal components, which may reveal potential patterns (IBM, 2020).

**FIGURE 2:**
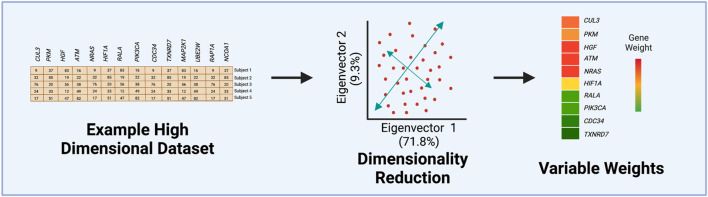
Steps of Principal Component Analysis (PCA). This example figure illustrates how the variance from an originating dataset, containing gene expression values, is compressed into the first eigenvectors by PCA. Often the first two eigenvectors are visualized to determine the percentage of the original variance able to be captured in the first two eigenvectors. The last step involves extracting quantified values for each variable’s contribution or weight to the first eigenvector.

PCA is routinely used for many data analysis applications in toxicology, including as a pre-processing step to identify potential sample outliers or to visualize clusters of variables or samples. In prior studies, PCA has been used to differentiate patient disease status such as hepatocarcinogen exposure groups (Furihata et al, 2016) and pulmonary arterial hypertension (Perez-Vizcaino et al, 2021). In a recent tobacco product use study from our group, k-means was first used to assign cytokine measures to different clusters, and then PCA was implemented to obtain an aggregate-level measure or eigenvector for each cluster (Payton et al, 2022). This approach permitted the comparison of cluster-based cytokine measures across tobacco product use groups, revealing greater statistical sensitivity when identifying exposure-induced changes in comparison to individual cytokine analyses (Payton et al, 2022). Overall, each of these examples highlights the value of implementing PCA in high-dimensional data analysis by reducing dimensionality, allowing for easier cluster visualization of a plethora of variables, and quantifying each predictor’s contribution to the principal components (Figure 2). For more examples and additionally methodologies surrounding unsupervised ML, see recent reviews (Omer et al, 2014; Kiselev et al, 2019; Verbeeck et al, 2020; Chaudhary and Singh, 2021).

## 4 Remaining challenges for analyzing high-dimensional data in toxicology research

The examples discussed highlight current issues at the intersection of data science and toxicology research where researchers are seeking to extract clinical and/or public health insight from a vast amount of data that are laborious and expensive to generate. This insight hinges upon the effective processing and analysis of data using techniques like data imputation, supervised ML, deep learning, unsupervised ML, and data reduction. All these methods have been pioneered in the fields of computer science and statistics, and so their adoption in our field is still relatively new. Moving forward it is imperative that more research be conducted to delineate which models are most applicable under various toxicology scenarios. These methods have limitations which are discussed further in the succeeding sections alongside questions posed for discussion amongst readers of this article.

### 4.1 Challenges in data pre-processing and ML

Limitations surrounding high-dimensional analysis include the following: There is insufficient documentation on toxicology-relevant high-dimensional analysis approaches. Information is often found in blogs and web pages rather than traditionally reputable sources like journal articles, which often omit analysis explanations and comparisons. Also, there is a general lack of sharing and reproducibility of trained algorithms between researchers. For data imputation specifically, it has the potential to bias downstream analyses, it is susceptible to skewed distributions, and can require large amounts of data to be accurate (Klein, 2022). As for ML, models are often difficult to interpret, with the underlying meaning and function sometimes referred to as a “black box” (Petch et al, 2022). Additionally, unsupervised ML can find correlations within data, but it does not mean that those correlations inherently have meaning or biological implications (IBM, 2020).

Based upon content provided throughout this perspective (above), as well as ongoing discussions between wet and dry lab scientists, several questions can be posed for further discussion:

• When should missing data be retained vs. discarded?
• Are there other methods to better incorporate missing data in toxicology?
• Are there specific ML models that work best for certain types of toxicological data?
• How can we better understand the biological meaning of ML-based findings?
• How can we better share predictive models built using data from one lab to predict outcomes using data in another lab?

### 4.2 Biases in data analysis approaches

It should be mentioned that any computational models we build are dependent upon the quality of the data, which often includes biases. Biases can occur when selecting which variables to collect and which features to include in the final model. It was once believed (and probably still believed) that algorithms inherently help address equity; however, data are often collected based on protocols/systems that have biases that are then perpetuated in any findings produced through high-dimensional data analyses. This belief was not likely held by researchers working specifically in social justice fields; however, the incorporation of a social justice and equity lens should be more consistently incorporated in toxicology research (Pettit, 2021). Artificial intelligence, an umbrella concept that includes machine learning (Hamet and Tremblay, 2017), has already become adopted in healthcare to predict disease outcomes, assess risk, and inform clinical decision-making. For example, previous clinical research has shown that medical decisions informed by models after adjusting for a covariate like race have both alleviated and exacerbated racial disparities (Foundation, 2021).

Effort is required to not perpetuate biases on all levels, which lead to the following questions for further discussion:

• Research group: is there sufficient diversity of ideas, experience, and background in the research group and the people specifically building the algorithms?
• Research questions: as toxicologists can we measure the impact of those social determinants of health by measuring epigenetic outcomes instead?
• Data collection: when gathering human data, what groups (i.e., demographic, exposure, etc.) are over or underrepresented?
• Algorithm training: if a predicted outcome is quantified, what variables have the largest impact on the model? What biases systemically or biologically need to be addressed as a result?
• Algorithm interpretation: is this model generalizable or interpretable enough to be useful on a similar dataset or context?

ML and artificial intelligence will continue to become more integrated into toxicological analyses as the prevalence of high-dimensional data continues to soar. We should learn from past mistakes of clinical applications of computational techniques and better design models that offer opportunities to extract insights while minimizing biases. First and foremost, as public health investigators, special attention and mention should be given to “the why” behind the models we build and their positive or negative implications on the communities we hope to serve.

### 4.3 High-dimensional data analysis training in toxicology

Advances in high-dimensional data analyses in toxicology will fail to evolve and advance over time if methods are not passed along to future leaders in the toxicology field. Hence, there is now an urgent need to generate and disseminate training materials surrounding high-dimensional data analyses. Our team has contributed to this need by developing the inTelligence And ML (TAME) Toolkit, which was organized to promote trainee-driven data generation, management, and analysis methods to “TAME” data in environmental health studies (Roell et al, 2022). Dissemination of these training materials has been and will continue to be organized through coursework and online workshops (Workshop, 2022), as well as a publicly available Bookdown site that guides participants through online training modules with underlying script and example datasets provided (UNC-SRP, 2023). Additional toxicology-relevant training materials and dissemination efforts are still needed worldwide and we believe these efforts are critical to the ongoing need to better handle high-dimensional data issues in our field. Although the methods highlighted in this article and TAME have been well documented in other fields like statistics, mathematics, and computer science, these materials serve to spark conversation and provide pertinent starting materials for wet bench scientists to incorporate analyses for large datasets especially those with high-dimensionality.

Given the current status of training resources for emerging scientists, we posed the following questions for further discussion:

• How can we more effectively train the next-generation of toxicologists on these methods?
• What are the best methods for reviewing trainee-driven analyses?
• Where are we still limited in data analysis training efforts in toxicology?
• How can we improve upon these methods as a concerted effort across toxicology groups?

## 5 Concluding remarks

As high-dimensional data become increasingly ubiquitous, the adoption of rigorous, standardized, high-throughput computing is imperative to extract novel insights. The advancement within the field of toxicology hinges upon leveraging computational techniques to better simulate what may potentially occur *in vivo* in conjunction with traditional methods that isolate a limited number of variables in analyses. These computational techniques, like data imputation and ML, help maximize the number of observations, predict exposure or disease outcomes, identify features that are most influential on an outcome, and reveal possible relationships between variables. Despite challenges regarding interpretability and health equity, effective data analysis approaches have the potential to yield novel toxicological results. These analyses will significantly advance the field of toxicology when emerging from continued interactions between wet and dry lab scientists.

## Data Availability

The original contributions presented in the study are included in the article/Supplementary Material, further inquiries can be directed to the corresponding author.
